# Irregular particle morphology and membrane rupture facilitate ion gradients in the lumen of phagosomes

**DOI:** 10.1016/j.bpr.2022.100069

**Published:** 2022-08-11

**Authors:** Maksim V. Baranov, Melina Ioannidis, Sami Balahsioui, Auke Boersma, Rinse de Boer, Manoj Kumar, Masato Niwa, Tasuku Hirayama, Qintian Zhou, Terrence M. Hopkins, Pieter Grijpstra, Shashi Thutupalli, Stefano Sacanna, Geert van den Bogaart

**Affiliations:** 1 Department of Molecular Immunology, Groningen Biomolecular Sciences and Biotechnology Institute, University of Groningen, Groningen, Netherlands; 2 Simons Centre for the Study of Living Machines, National Centre for Biological Sciences, Tata Institute of Fundamental Research, Bangalore, India; 3 Laboratory of Pharmaceutical and Medicinal Chemistry, Gifu Pharmaceutical University, 1–25–4, Daigaku-nishi, Gifu 201–1196, Japan; 4 ^I^nternational Centre for Theoretical Sciences, Tata Institute of Fundamental Research, Bangalore, India; 5 Molecular Design Institute, Department of Chemistry, New York University, New York, NY, United States; 6 Department of Medical Biology and Pathology, University Medical Center Groningen, Groningen, Netherlands

## Abstract

Localized fluxes, production, and/or degradation coupled to limited diffusion are well known to result in stable spatial concentration gradients of biomolecules in the cell. In this study, we demonstrate that this also holds true for small ions, since we found that the close membrane apposition between the membrane of a phagosome and the surface of the cargo particle it encloses, together with localized membrane rupture, suffice for stable gradients of protons and iron cations within the lumen of the phagosome. Our data show that, in phagosomes containing hexapod-shaped silica colloid particles, the phagosomal membrane is ruptured at the positions of the tips of the rods, but not at other positions. This results in the confined leakage at these positions of protons and iron from the lumen of the phagosome into the cytosol. In contrast, acidification and iron accumulation still occur at the positions of the phagosomes nearer to the cores of the particles. Our study strengthens the concept that coupling metabolic and signaling reaction cascades can be spatially confined by localized limited diffusion.

## Why it matters

Molecular crowding by organelles and macromolecules limits the diffusion of biomolecules. Using micrometer-sized hexapod-shaped particles that mimic the morphology of fungal pathogens, we show that the diffusion of ions is also limited within the lumen of phagosomes. At the sharp tips of the particles, protons and iron leak from the phagosomes into the cytosol. However, iron accumulation and acidification still occur at the particles’ cores, resulting in relatively stable gradients. These findings provide an important new insight into the phagocytic processing of differently-shaped pathogens by immune cells: that the close apposition between the membrane of phagosomes and the surface of cargo particles limits diffusion even of small ions.

## Introduction

Microbial spread is actively contained by phagocytic cells of the mammalian immune system, especially neutrophils, macrophages, and dendritic cells. These cell types can engulf pathogenic microbes and non-microbial particles, such as monosodium urate crystals in gout, cholesterol crystals in atherosclerosis, and hydroxyapatite crystals in osteoarthritis, through phagocytosis. This process is initiated by plasma membrane extensions, so-called pseudopods, that extend around the phagocytic cargo and trap it into a membrane-bound organelle called a phagosome (1,2). Following their formation, nascent phagosomes undergo maturation by a series of membrane fission and fusion events with endosomes and lysosomes. This maturation is characterized by the disappearance of early endosome antigen 1 (EEA1) from the membrane of the phagosome, the acquisition of lysosomal-associated membrane protein 1 (LAMP1) (3), and an increase in the luminal iron content (4,5). The fusion with lysosomal compartments also results in the acquisition of the v-ATPase (vacuolar proton ATPase) to phagocytic membranes (6). The v-ATPase acidifies the phagosomal pH by pumping protons from the cytosol into the phagosomal lumen (7), which is a prerequisite for efficient microbial killing, antigen degradation, and presentation.

Overall, it is widely appreciated that the shape of microbial pathogens and other phagosomal cargoes can strongly affect phagosomal physiology, including the rate of phagocytosis, downstream immune responses, and cell death (2). For example, recent findings show that the fungal pathogen *Candida albicans* prevents acidification by changing from spherical yeast-like to extended hypha-like morphologies that rupture the phagosomal membrane, which leads to the leakage of protons out of the phagosomal lumen into the cytosol (7,8). However, pathogenic targets vary widely in shape and produce virulence factors, making it hard to draw general conclusions on how their morphology affects phagosomal maturation. To overcome this problem, experiments have been performed with model particles with precisely defined shapes (2). However, these particles are mostly of small (<1 μm) size (2), smaller than the size of fungal pathogens, and are challenging to resolve with optical microscopy (1).

In this study, we engineered large (>6 μm) hexapod-shaped particles to mimic hyphenated fungal pathogens for studying phagosomal physiology. We observed the localized rupture of the phagosomal membrane at the tips of the hexapods, accompanied by the localized recruitment of the cellular repair protein galectin-3 to the phagosomal membrane (9,10). We found that the lumen of the phagosomes did not acidify at the position of the tips of the particles. In contrast, acidification occurred at the core of the particles, which is surprising given the extremely fast diffusion of protons in aqueous environments. The luminal iron content showed a similar gradient, and iron was accumulated at the cores but not at the tips of the phagosomes. Electron microscopy showed close apposition between the particle surface and the membrane of the phagosome. These data demonstrate that the activity of the v-ATPase, the close apposition of the phagosomal membrane with the surface of an irregularly shaped phagosomal cargo particle, and localized membrane rupture suffice for the generation of stable ion gradients within the phagosomal lumen. Our findings have implications for our understanding of the signaling of small second messengers and the coupling of metabolic reaction cascades in highly crowded intracellular environments.

## Results

### Phagocytosis of hexapod-shaped particles

**Figure 1:**
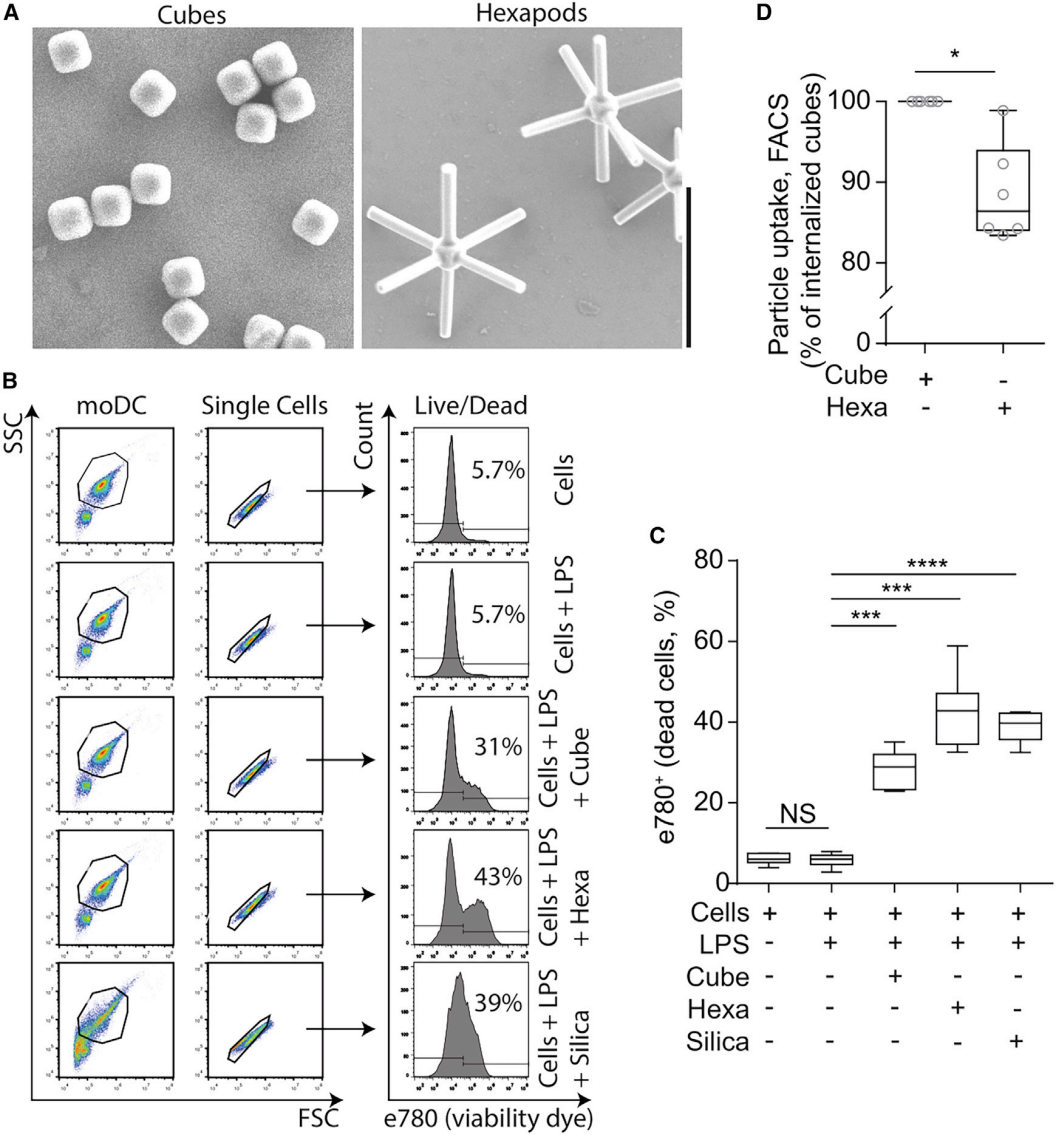
Silica hexapod-shaped particles are cytotoxic. (*A*) Scanning electron microscopy (SEM) of silica cubes and silica hexapods. Scale bar: 5 μm. (*B*) Flow cytometry gating strategy plots representing side scatter (SSC) and forward scatter (FCS) of monocyte-derived DCs (moDCs) phagocytosing either cubes or hexapod-shaped particles and corresponding viability as measured with e780 dye. (*C*) Quantification of (*B*). (*D*) Percentage of cube or hexapod particle uptake normalized to cube uptake from (*B*). Statistics: (*C*), paired *t*-test; (*D*), non-parametric paired *t*-test.

Fungal pathogens such as *C. albicans* have micrometer-long protrusive hyphens that extend from a spherical body (7). To mimic this extreme shape, hexapod-shaped colloidal particles were synthesized. Hexapod colloids consist of cubic hematite/silica core (∼1 μm) and uniformly grown silica rods (∼2.5 μm in length) emanating from each of the six faces of the cube (11). Silica cubes without rods were used to mimic spherical, non-hyphenated yeast particles (Fig. 1 *A*). Dendritic cells were derived from monocytes of healthy human blood donors (moDC), a highly phagocytic cell type (12), and co-cultured with either the cubes or the hexapods. Silica particles are well known to induce cytotoxicity (2,13,14), and approximately ∼30%–40% of the cells died after 5 h in culture with both the cubes and the hexapod-shaped particles, similar to silica chips (Fig. 1 *B* and *C*). Overall, particles with complex morphologies are ingested less efficiently than spherical particles, due to the higher membrane deformation energy cost required for their uptake (2). Indeed, the phagocytic efficiency of the hexapods was ∼17% lower than of the cubes (Fig. 1 *D*). Phagosomal maturation was observed for phagosomes containing both particle shapes, as LAMP1-positive phagosomes were observed both for the ingested hexapods and cubes (Supporting Material Fig. 1 *A* and *B*) and the intensity of LAMP1 recruitment was similar for both particle types (Fig. S1 *C*).

### Phagosomes carrying hexapod-shaped particles have pH gradients

**Figure 2:**
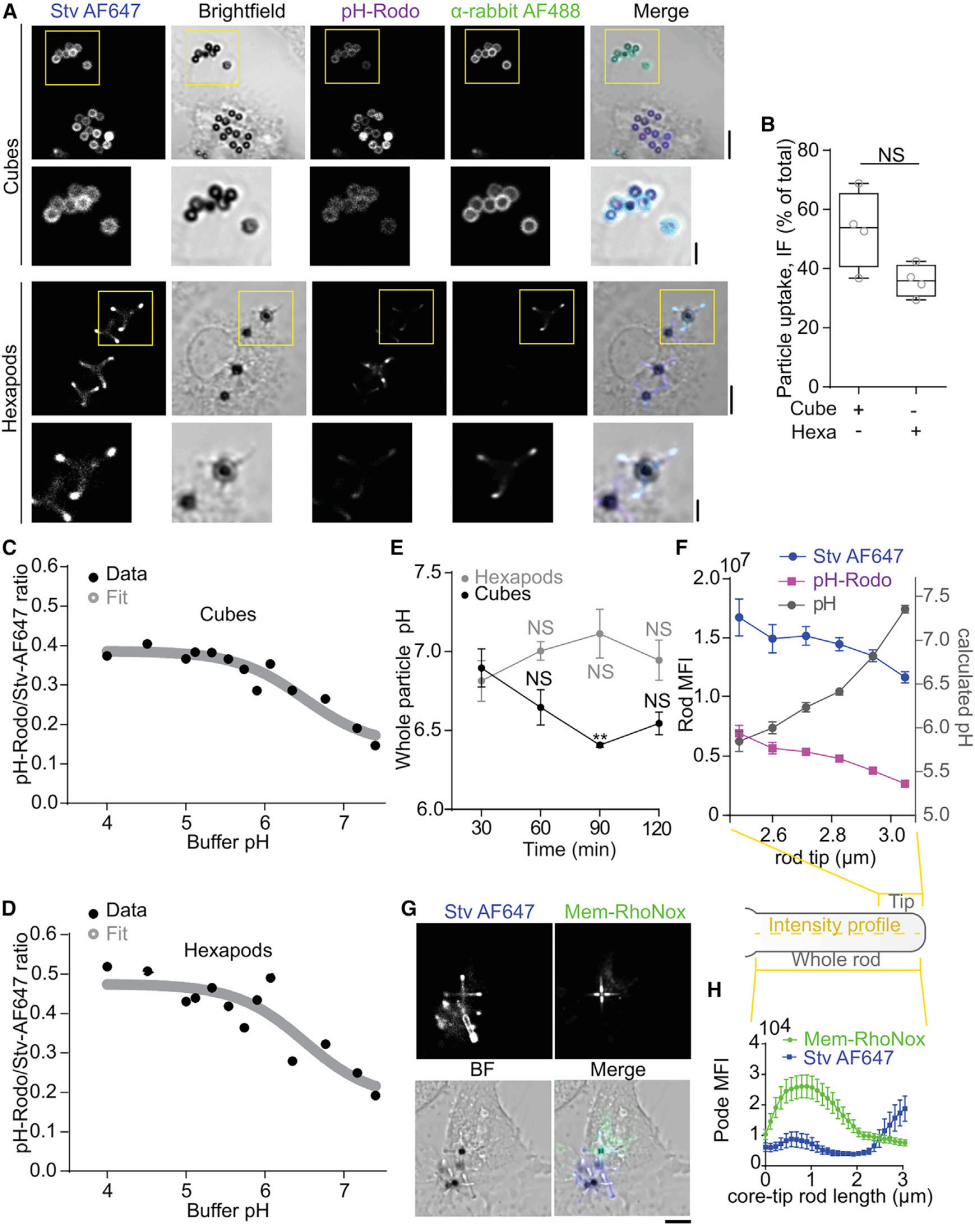
Phagosomes containing hexapod-shaped particles do not acidify. (*A*) Representative confocal micrograph of monocyte-derived DCs (moDCs) pulsed with streptavidin-Alexa Fluor 647 (Stv AF647, blue in merge) and avidin-pHRodo-red (pHRodo, magenta)-labeled silica cubes (upper) and hexapods (lower). Anti-streptavidin-rabbit-antibody was used for particle opsonization to facilitate the Fc-mediated uptake; α-rabbit antibody was used to label un-internalized particles (α-rabbit AF488, green). (*B*) Quantification of particle uptake from (*A*). (*C*) Calibration curve and fit (gray) of pHrodo/Stv-AF647 ratios (mean ± SEM, ∼100 cubes per buffer). (*D*) The same as (*C*) but for hexapods. (*E*) pH for phagosomes carrying cubes (black) or hexapods (gray) in moDCs from four donors pulsed for the indicated times (mean ± SEM; automated analysis (see Supporting Material Fig. 3*A*) based on ∼112 particles/per donor/time point). (*F*) Intensity plot profile of 0.5-μm hexapod tip: Stv AF647 (blue), avidin-pHRodo-Red (magenta), and corresponding pH values (gray). (*G*) Representative confocal micrographs showing Mem-RhoNox (green) membrane staining (fluorescent in presence of labile Fe(II); green in merge) in moDCs ingesting hexapods. (*H*) Intensity plot profiles per rod on images from G (mean ± SEM; ∼30 rods). Scale bars: main images, 5 μm; insets, 2 μm. Statistics (*E*), one-way ANOVA, all significance is calculated in relation to the 30 min time point; (*B*), paired *t*-test.

To compare the acidification of phagosomes carrying cubes and hexapods, the silica surface of the particles was first functionalized with biotin and then labeled with the pH-sensitive probe avidin-pHRodo (Figs. 2 *A* and S2A). As a reference marker, the particles were also labeled with streptavidin conjugated to the pH-insensitive fluorophore Alexa Fluor 647 (stv-647). Likely due to non-uniform surface chemistry, the pHRodo and stv-647 signals were substantially brighter at the tips and could be used for identifying the tip positions in confocal microscopy imaging (Figs. 2 *A* and S2A). To improve the uptake efficiency, the particles were opsonized with rabbit immunoglobulin (Ig) G raised against streptavidin, which facilitates Fc-receptor-mediated uptake by human macrophages and DCs (15) (Figs. 2 *A* and S2A). This opsonization had another advantage, because it facilitated the discrimination between completely ingested particles from non or incompletely phagocytosed particles using a fluorescently labeled secondary antibody raised against rabbit IgG (Fig. 2 *B*).

To generate a pH calibration curve, we determined the ratios *R* between the pHRodo and stv-647 fluorescence intensities from confocal microscopy images of particles in buffers with pH values ranging between 4 and 7.7 for both cubes and hexapods (Figs. 2 *C*, *D*, S2 *B*, and *C*). The calibration data were fitted with:

(1) $$pH=\;-\text{log}\left(\frac{K_{a}BR}{A-BR}\right),$$

with *R* assumed to be linearly dependent on the protonated form of pHRodo with factor *B*, *A* the maximum amount of pHRodo, and *K*_a_ the acid dissociation constant of pHRodo (pKa = 6.5).

Next, human moDCs were pulsed with cubes or hexapods. Live-cell microscopy revealed uptake of the particles over time (Video S1). Confocal microscopy images were acquired at 30, 60, 90, and 120 min after particle addition (Figs. 2 *E*, S2 *D*, and *E*). The ratios between the pHRodo and stv-647 signals were then converted into pH values by comparison with the standard calibration curves (Fig. 2 *C* and *D*). Automated analysis on whole particles (Fig. S3 *A*) showed that phagosomes containing cubes readily acidified over time, reaching pH ∼6 after 90–120 min (Fig. 2 *E*). Note that this is an upper estimate, as we cannot detect lower pH values with pHRodo. In contrast, phagosomes containing hexapods did not show apparent acidification and retained approximately an overall neutral pH (Fig. 2 *E*). However, we noticed that phagocytosed hexapods displayed a clearly increased pHRodo signal along the length of the rods, but less at the tips (Fig. 2 *A* and S2E). This heterogeneity was not observable when the particles were directly submerged in buffers with high acidity (Fig. S2 *C*), suggesting spatial heterogeneities in pH in the lumen of phagosomes. Conversion of the pHRodo/stv647 ratios along the rods into pH values (Fig. S3 *A*) showed that the pH was approximately pH 6 at 0.5 μm distance away from the tips, while it approximated neutral pH at the tips (Figs. 2 *F* and S3B).

Video 1. Live imaging of hexapod uptake by moDCs over 5 h. Cyan, DAPI; green, hexapod-labeled stv-647

Phagosomes are expected to acidify less in dendritic cells compared with macrophages, because protons are sequestered by superoxide anions produced by the NADPH oxidase NOX2 (16) and/or NOX2-produced radicals rupture the endosomal membrane (17). We therefore also pulsed human peripheral blood monocyte-derived macrophages with the pHRodo and stv647-labeled hexapods. However, we obtained similar results with macrophages as with the moDCs: absence of significant acidification of phagosomes carrying hexapods and acidification predominantly visible near the cores of the hexapods (Fig. S4).

### Phagosomes carrying hexapod-shaped particles have iron gradients

As an independent confirmation of the heterogeneity of the luminal contents of phagosomes carrying hexapod-shaped particles, we measured the content of phagosomal iron in moDCs using the lipophilic probe Mem-RhoNox (18). Mem-RhoNox inserts in cellular membranes, and its fluorescence signal correlates with the content of iron (18). Phagosomes display elevated iron concentrations upon their fusion with lysosomes, as lysosomes are the main pool of free iron in the cell (4). Similar to our pH observations, we observed a clear signal of Mem-RhoNox along the rods but not at the tips of the rods (Fig. 2 *G* and *H*).

**Figure 3:**
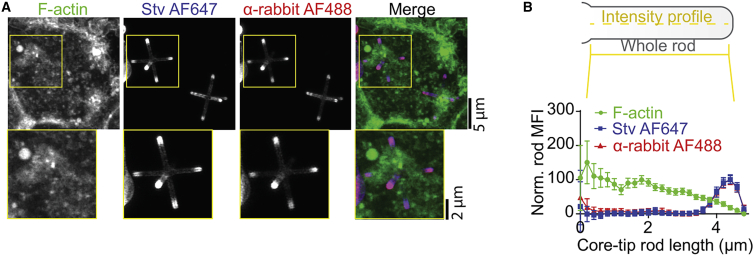
Phagosomes containing hexapod-shaped particles show less F-actin at tips. (*A*) Representative confocal micrograph of moDCs pulsed with streptavidin-Alexa Fluor 647 (Stv AF647, blue in merge)-labeled hexapods. Anti-streptavidin-rabbit-antibody was used for particle opsonization to facilitate the Fc-mediated uptake; α-rabbit antibody was used to visualize the anti-streptavidin-rabbit-antibody distribution (α-rabbit AF488, red). F-actin was labeled with phalloidin (green). (*B*) Intensity plot profiles of F-actin and streptavidin-AF647 at rods from (*A*) (mean ± SEM; ∼128 rods). Scale bars: main images, 5 μm; insets, 2 μm.

Additionally, we observed an F-actin exclusion from the rod tips, possibly due to a loss of membrane-anchored F-actin at the tips (Fig. 3; Video S2).

Video 2. Three-dimensional reconstruction of confocal imaging of a hexapod (stv-647, green) internalized by a monocyte-derived dendritic cell. Red, F-actin; gray, DAPI

### Localized membrane rupture at the tips of hexapod-containing phagosomes

**Figure 4:**
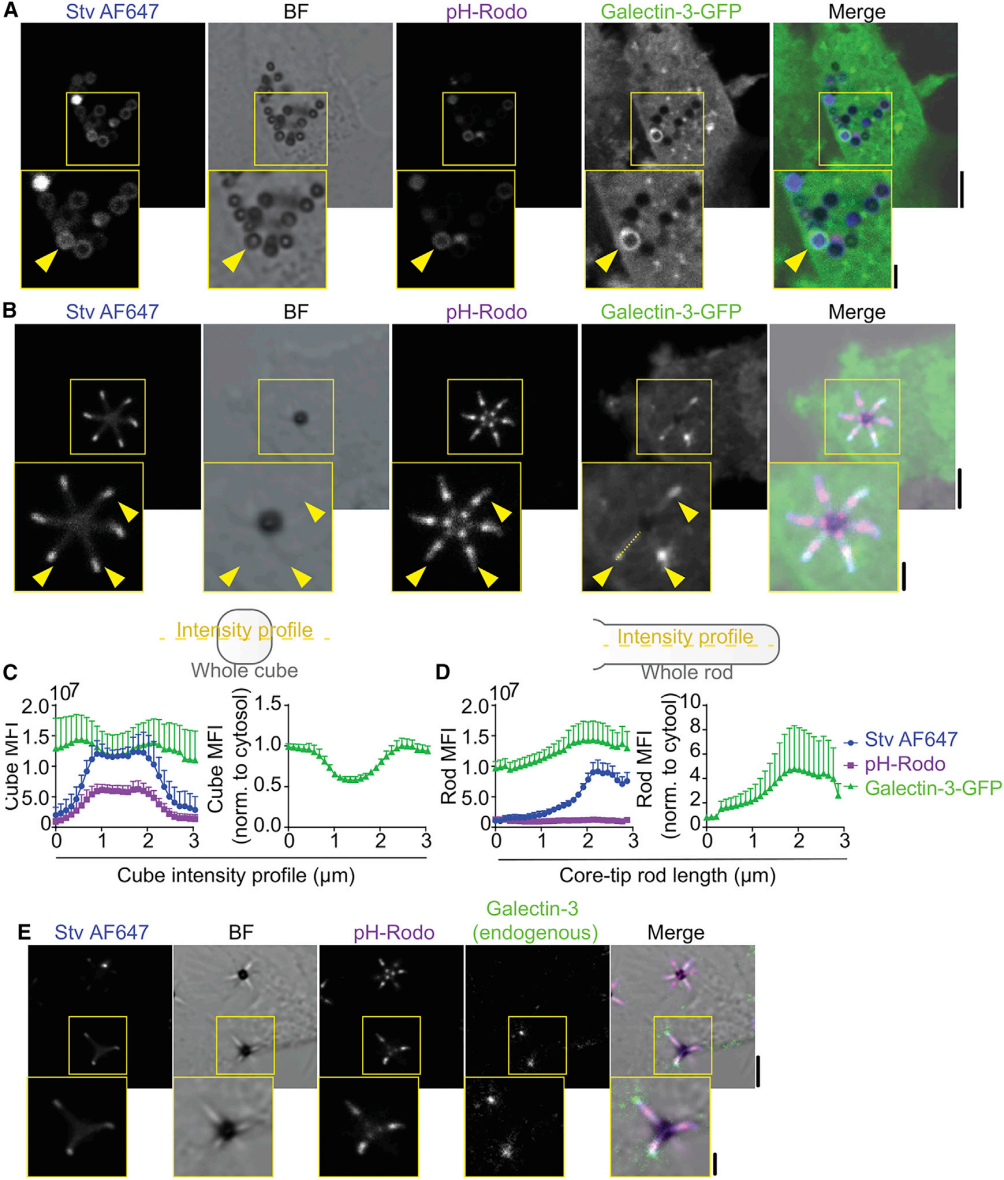
Localized phagosomal membrane rupture at tip of hexapod-shaped particles. (*A*) Representative confocal micrograph showing overexpression of galectin-3-GFP (green) in moDCs followed by stimulation with cube particles for 120 min. (*B*) Same as (*A*) but for hexapods. (*C*) Intensity cross-section profile per cube (left-hand side) and normalization to cytosolic background (mean per donor ± SEM; ∼40 cubes/per donor; four donors) (*D*) Same as (*G*) but intensity plot profiles over the length of the rods of the hexapods (mean per donor ± SEM; ∼34 hexapods/per donor; four donors). (*E*) Representative confocal micrograph showing immunolabelling of endogenous galectin-3 (green in merge) in moDC stimulated with hexapods for 120 min. BF, bright-field. Scale bars: main images, 5 μm; insets, 2 μm.

We reasoned that the heterogeneity of the pH and iron contents in the lumen of hexapod-containing phagosomes would be caused by localized rupture of the phagosomal membrane at the tips of the hexapods. Silica particles are known to cause rupture of phagosomal membranes (19), which leads to a cascade of repair mechanisms, such as budding, endocytosis, and patching for membrane closure (20). The rupture of phagosomal membranes results in the recruitment of galectin-3 to glycosylated proteins and lipids exposed in the cytosol (8,21,22). In intact phagosomes, these glycosylated proteins and lipids only face the phagosomal lumen and are inaccessible to cytosolic galectins. However, they become accessible upon loss of integrity of the phagosomal membrane due to diffusion of galectin into the phagosomal lumen and/or the translocation or flipping of glycosylated proteins and lipids to the endocytic membrane leaflet, resulting in the localized recruitment of galectin-3 (8,13,21,22). We overexpressed galectin-3 fused with a green fluorescent protein (GFP) (23) in moDCs, and pulsed them with the silica chips (Fig. S5 *A*), hexapod, or cube-shaped particles (Fig. 4 *A* and *B*). In galectin-3-GFP expressing cells, the profiles of GFP intensity at cubes or along the hexapod rods were plotted relative to the cytosolic levels (Fig. 4 *C* and *D*). Although phagosomes carrying cubes were mostly negative for galectin-3 and the fluorescence observed at cubes was similar to the cytosolic background level (Fig. 4 *C*, right panel), by far most phagosomes carrying hexapods demonstrated steadily increasing levels of galectin-3 specifically at the tips, sometimes reaching 4–15 times higher signal compared with cytosolic levels (Fig. 4 *D*, right panel). Similarly, endogenous galectin-3 also accumulated at the tips of hexapod-containing phagosomes, whereas we did not observe localization to cube-containing phagosomes (Fig. 4 *E* and S5 *B*–*D*). These findings indicate that the “pointy” geometry of hexapods was more likely to cause phagocytic membrane rupture specifically at the tips of the rods.

**Figure 5:**
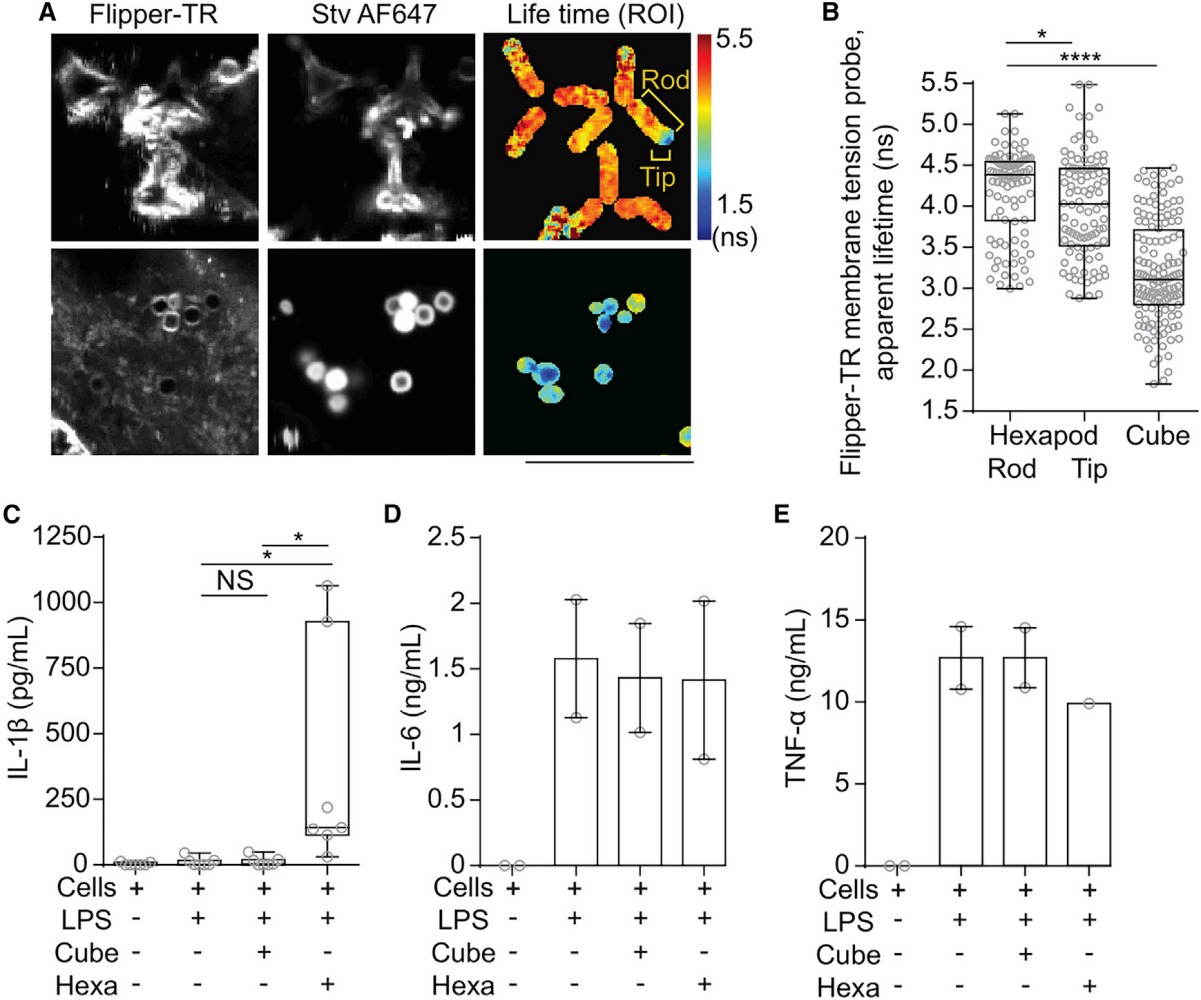
Particle geometry affects membrane tension and hexapod particles induce IL-1β production. (*A*) Representative confocal microscopy and fluorescence lifetime imaging microscopy (FLIM) images of moDCs pulsed with streptavidin-Alexa Fluor 647 (Stv AF647)-labeled hexapods (top row) or cubes (bottom row). Cells were labeled with Flipper-TR membrane tension probe. ROI, region of interest. Scale bar, 5 μm. (*B*) Quantification of (*A*) (∼140 cubes and ∼97 rods). Statistics: comparison of rod body with tips, paired *t*-test; rod body with cubes, unpaired *t*-test. (*C*) IL-1β levels in supernatants from cells of moDCs pre-treated for 2 h with LPS, followed by 3 h of incubation with the particles (experiment repeated in seven donors). (*D*) IL-6 production in supernatants from (*E*) (two donors). (*E*) Same as (*D*) but for TNF-α. Statistics: (*C*–*E*), one-way ANOVA.

The Flipper-TR probe inserts in biological membranes and has a fluorescent lifetime that depends on the lateral tension within the membrane (24). Thereby, membrane tension can be measured with fluorescence lifetime imaging microscopy (FLIM). The fluorescence lifetime of the Flipper-TR probe decreased at the tips of phagosomes containing hexapod particles compared with along the rods, strengthening our conclusion that the membranes are ruptured at the tips. However, we detected even lower membrane tension at cube-containing phagosomes, suggesting that the particle geometry affects membrane stretching and tension (Fig. 5 *A* and *B*).

### Hexapods promote the production of interleukin-1β

Phagosomal membrane rupture can result in leakage of K^+^ and hydrolytic proteinases, such as cathepsin B, from the lumen of phagosomes (14,25). This in turn can result in activation of the NLRP3 inflammasome, leading to conversion of pro-interleukin (IL)-1β to mature IL-1β (8,25, 26, 27). Moreover, shape-dependent inflammasome activation upon phagosome rupture has been shown previously to result in inflammasome activation, as the transition of the intracellular pathogen *C. albicans* from its yeast to its hyphal form results in inflammasome activation and IL-β production in macrophages (28). To test if the rupture of hexapod-containing phagosomes would also lead to IL-1β production, moDCs were pulsed either with cubes or hexapods. In addition, the cells were stimulated with the pathogenic stimulus endotoxin (lipopolysaccharide (LPS)), which promotes transcription of the gene coding for pro-IL-1β. As anticipated, we observed a significant increase in IL-1β production for hexapods but not for cubes (Fig. 5 *C*). As expected, the production of inflammasome-independent cytokines IL-6 and tumor necrosis factor (TNF)-α were not affected by the particle type, and they were produced at comparable levels in all cells stimulated with LPS (Fig. 5 *D* and *E*).

### Close apposition between phagosomal membrane and hexapod surface

**Figure 6:**
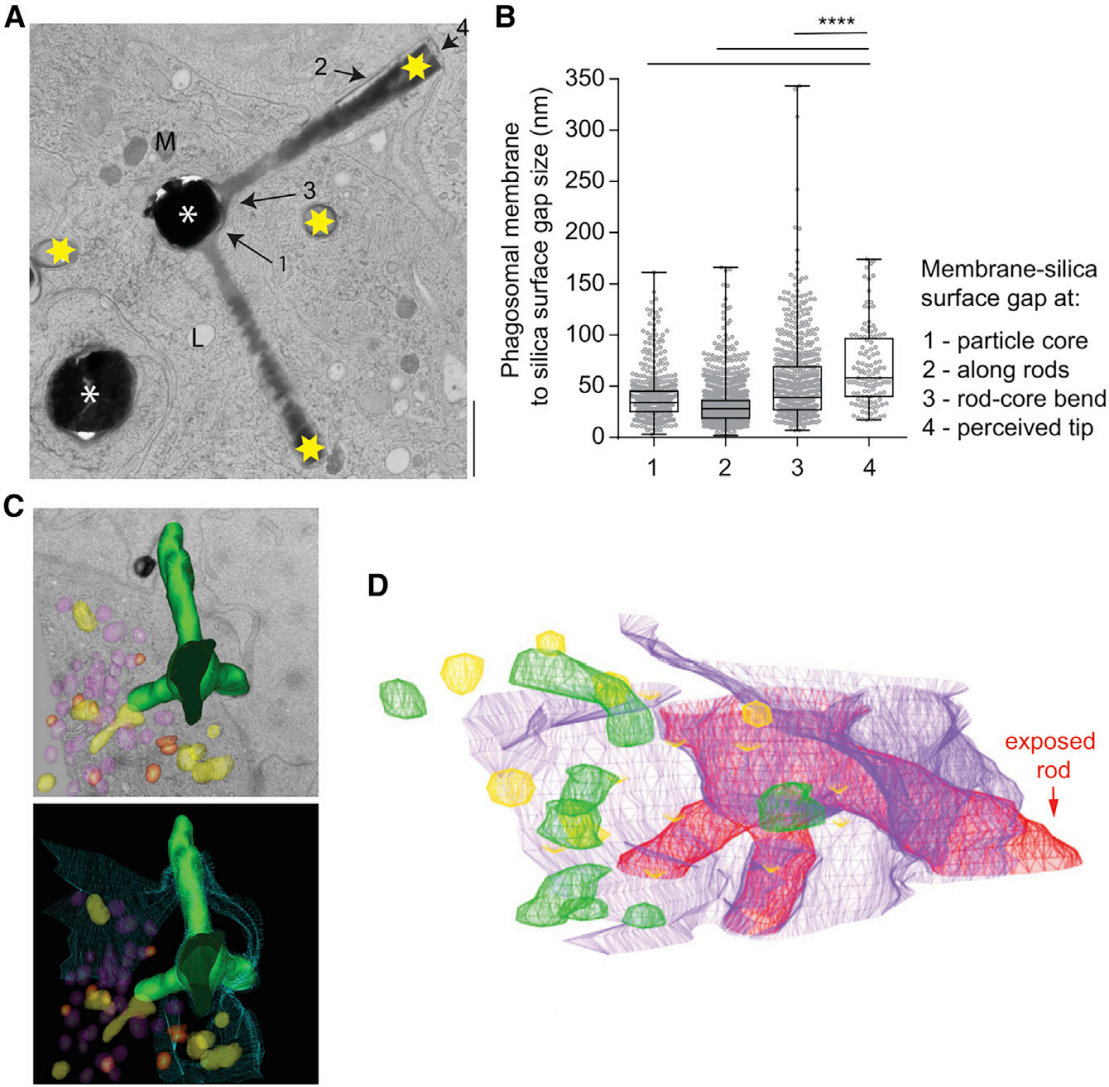
Phagocytic membranes rupture at the tips of hexapods. (*A*) Transmission electron microscopy of a cryofixed moDC with a phagosome carrying a hexapod. White stars, hexapod iron-based cores; yellow stars, hexapod tips; black arrows, different regions on a hexapod used for estimating the distance between the silica surface and phagocytic membrane cores. Scale bar, 1 μm. (*B*) Quantification of membrane distances as indicated in (*A*). (*C*) Three-dimensional reconstruction of the electron-microscopy images from serial sections of the silica surface (green) and vesicular structures (yellow, purple, red). (Lower) Plasma membrane (blue mesh) continues around the silica hexapod (green). (D) Three-dimensional reconstruction of the electron-microscopy images from (*C*). Red, hexapod; purple, plasma membrane still continues invaginating and around the hexapod; green, mitochondria; yellow, endo/lysosomes. Exposed rod is shown with arrow.

We reasoned that the gradients of protons and iron would only be possible if the phagosomal membrane was closely apposed to the surface of the particles, thus limiting diffusion. Indeed, serial sectioning transmission electron microscopy of moDCs containing hexapod-containing phagosomes revealed that these particles are tightly sequestered by phagocytic membranes (Fig. 6 *A*, *C*, and *D*; Videos S3 and S4). To quantify the gap size, we measured the distance between the phagosomal membrane and the particle surface at various positions of the particles: along the rod, at the perceived tips (i.e., the imaged end of the rod, either the tip or cross section of the rod), and at the core (Fig. 6 *B*). An average gap size was approximately 20–35 nm, and the smallest gap size was observed along the rods, while the largest gap size was observed at the perceived tips, possibly due to the large curvature of the particle and/or the membrane rupture at these positions. Although the difference in membrane apposition might affect the measurements of ion gradients, we consider this unlikely, as the gradients were observed both for protons with pHRodo conjugated to the particles, and for iron with Mem-RhoNox inserted in the phagosomal membranes. Moreover, we also observed close membrane apposition in rare cases when a particle was not yet completely ingested (see Video S4 for an example of three-dimensional (3D) electron microscopy reconstitution).

Video 3. Three-dimensional reconstruction of an electron microscopy serial section images of a hexapod (red) internalized by a monocyte-derived dendritic cell. The phagosome is surrounded by intracellular organelles: mitochondria (green), endo/lysosomes (yellow), and nucleus (blue)

Video 4. Three-dimensional reconstruction of an electron-microscopy imaging of a hexapod (red) internalized by a monocyte-derived dendritic cell. The phagosome is surrounded by intracellular organelles: mitochondria (green), endo/lysosomes (yellow), and plasma membrane (purple). Note the incomplete internalization of the particle

## Discussion

It is known that intracellular pathogens use different strategies to evade or adapt to the bactericidal environments in phagosomes, and they can dynamically adjust their metabolism or produce virulence factors that help them escape host cell defense mechanisms (6). To dissect the effects of the pathogen shape, we generated hexapod-shaped particles of comparable size as hyphenated (hexapods) or non-hyphenated (cubes) fungal pathogens. Our data show that the membrane of the phagosome carrying hexapod-shaped particles ruptures at the tips of the rods but not at other positions. This localized membrane rupture seems to be reminiscent of the rupture induced by hyphenated fungal pathogens, particularly *C. albicans*. This pathogen survives in phagosomes by expanding and rupturing the phagosomal membrane, causing proton efflux from the phagosomal lumen (7,8). Our data show that, although the phagosome no longer acidified at the tips of the hexapod-shaped particles, we still observed phagosomal acidification and iron accumulation near the cores, which are hallmarks of phagosomal maturation. Moreover, fusion with lysosomal compartments still occurred, as we observed recruitment of LAMP1. This LAMP1 recruitment to phagosomes could be expected, as it is independent of the v-ATPase (29).

Our data show that the localized leakage at the tips of hexapod-containing phagosomes leads to heterogeneity in the contents of protons and metal irons within the lumen of the phagosomes. While membrane leakage occurred at the tips, acidification and iron accumulation could still be observed near the core of the particles. These findings reveal a very slow effective diffusion of protons and iron within the aqueous volume of phagosomes coupled to a high proton and iron import. A small, effective volume might partly explain this slow diffusion in the phagosomal lumen. Indeed, our electron microscopy reveals a close membrane apposition between the surface of the particle and the membrane along the rods. Although this distance might be affected by artifacts from the electron microscopy, for example, the EPON embedding and particularly the microtome slicing of EPON resin embedded samples. In addition, the effective volume might be confined by crowding of biomacromolecules, particularly by the glycocalyx at the luminal surface of the phagosomal membrane. Glycolipids and glycoproteins extend long and branched polysaccharides into the interior of the phagosome, which are relatively immobile and might hinder diffusion. Such confined volumes can have a direct effect on rates of diffusion. For example, proton diffusion can be slowed down by 100 times if a water droplet diameter changes from 5 nm to <1 nm (30). Moreover, lateral proton diffusion along lipid bilayers was estimated to occur over long distances in water (100 μm), but this was significantly impeded by physiological buffer concentrations leading to a proton diffusion distance of only 10 nm (31). Of course, we expect that not only the diffusion of protons and iron is very slow in the particle-containing phagosomes but also of other ions and other molecules, like metabolites and proteins.

Our finding that close membrane apposition in phagosomes carrying irregularly shaped targets can lead to a substantially slower diffusion, even of protons and other ions, has an impact on our understanding of cellular physiology, particularly the signaling from ions and small second messengers. For example, in calcium-mediated neurotransmitter release, it is well understood that the influx of calcium through channels localized at the presynaptic membrane leads to a transient and highly localized elevation of calcium at the presynaptic fusion site. These calcium channels are not localized randomly but are juxtaposed to the synaptic fusion sites, and this is required for calcium-mediated neurotransmitter release. It is proposed that the channel–vesicle distance may vary between 30 and 40 nm or even >200 nm for efficient neurotransmitter release (32,33). However, smaller distances of about 20 nm away from the calcium channel allow for higher localized Ca^2+^ saturation per unit of time, making it less susceptible to chelating buffers (32). Given the high organellar and macromolecular crowding at synaptic boutons (34), our findings might aid a quantitative understanding of the magnitude and localization of these localized calcium spikes. A second example is that molecular crowding might contribute to cellular heterogeneities of cyclic AMP (cAMP), which mediates highly localized cellular events. The cAMP concentration is not uniform within cells and it is not controlled by simple diffusion. Inactive cAMP accumulates in nano-domains of 10 nm in diameter and diffuses very slowly. However, upon external stimulation, cAMP rapidly becomes unbound or accessible for downstream signaling, and this buffered slow diffusion of cAMP creates conditions for fine-tuned localized signaling (35).

Our study demonstrates that ∼6-μm-sized hexapod-shaped particles can be used to gain insights into how a pathogen’s morphology affects the physiology of phagosomes, and membrane tension, repair, antigen leakage, and antigen presentation can be studied in a precise and reproducible manner (2). These particles are a useful tool to study metal transport and acidification, membrane integrity, and its link to cytoskeletal reorganization. They might also have potential therapeutic applications, as the membrane rupturing by nanoparticles might be used for drug delivery (36) or to improve the immunogenicity of nanoparticle vaccination (14). Mesoporous spherical (37, 38, 39, 40, 41, 42) as well as rod-shaped (43) silica particles are used as platforms for intracellular drug delivery. Silica nanoparticles with mesoscale porosity and virus-like size and nano-scale spikes have been proved to be promising drug-delivery systems in anti-cancer therapies (44).

## Materials and methods

### Antibodies and reagents

Streptavidin-Alexa Fluor 647 conjugate (S21374), pHRodo Red avidin (P35362), DQ-OVA (D12053) and 568 phalloidin Alexa Fluor 488 (A12380) were purchased from Thermo Fisher. Silane-PEG-biotin (PG2-BNSL-3k-100 mg, used at 6 mg/ml) and Silane-PEG-NHS (PG2-NSSL-5k-100 mg) were purchased from Nanocs. The following primary antibodies were used: anti-streptavidin rabbit polyclonal IgG (Novus Biologicals, NB100-1675-1 mg), anti-mouse/human Mac-2 (Galectin-3) rat monoclonal IgG2a (Cedarlane, CL8942AP; used at 1:1000), and anti-human CD107a (LAMP1) (BioLegend, 328602; used at 1:200). The following secondary antibodies were used at 1:400 dilution: goat anti-mouse IgG1 Alexa Fluor 647 (A-21240), goat anti-rat IgG (H+L) Alexa Fluor 568 (A-11077), donkey anti-goat IgG (H+L) Alexa Fluor 647 (A-21447), donkey anti-rabbit IgG (H+L) Alexa Fluor 647 (A-31573), goat anti-rabbit IgG (H+L) Alexa Fluor 568 (A-11011), donkey anti-rat IgG (H+L) Alexa Fluor 488 (A21208), donkey anti-mouse IgG (H+L) Alexa Fluor 647 (A-31571), and donkey anti-mouse IgG (H+L) Alexa Fluor 488 (A21202); all purchased from Thermo Fisher.

Human IL-1β (Thermo Fisher; 88-7261-88), IL-6 (Thermo Fisher; 88-7066-88), and TNF-α (Thermo Fisher; 88-7346-88) were measured by ELISA following the manufacturer’s kit guidelines using 96-well flat-bottom plates (Greiner bio-one Microlon; 655092).

Mem-RhoNox was used for intra-phagosomal detection of the labile Fe(II) ions following the protocol described in (45). In short, the moDCs were pre-cultured for adherence for 1 h in a glass-bottom Ibidi plate (80827) in serum-free phenol-red-free RPMI containing 1% glutamine. Next, the cells were stimulated with particles for 30 min, followed by the medium exchange containing 1 μM Mem-RhoNox for 15 min at 37°C 5% CO_2_. Next, the cells were washed twice with fresh pre-warmed medium and imaged with a confocal microscope (excitation 546 nm, emission 568 nm).

### Generation of moDCs and macrophages

Peripheral blood mononuclear cells (PBMCs) were isolated from buffy coats of healthy blood donors. Adherent monocytes were separated from leukocytes based on their adherence to a T75 culture flask. For further differentiation into moDCs, the monocytes were cultured for 6 days in complete RPMI-1640 medium (Gibco, 32404-014) containing 10% fetal bovine serum (FBS) (Thermo Fisher, 10309433), 1% L-glutamine (Gibco, 21875-034), and 1% antibiotic-antimitotic (Gibco, 15240062), supplemented with 300 U/ml IL-4 (Miltenyi, 130-093-924) and 450 U/ml granulocyte-macrophage colony-stimulating factor (GM-CSF) (Miltenyi,130-093-867), at 37°C 5% CO_2_ (46). Macrophages were differentiated with the same protocol, but now using 100 ng/ml macrophage colony stimulating factor (M-CSF) (R&D Systems, 216-MC). Approval to conduct experiments with human blood samples was obtained from the blood bank, and all experiments were conducted according to national and institutional guidelines. Informed consent was obtained from all blood donors by the Dutch blood bank. Samples were anonymized and none of the investigators could ascertain the identity of the blood donors.

### Generation of the cube and hexapod particles

Hematite microcubes were prepared via a sol-gel method as described in (47). Briefly, a 100-ml aqueous solution of FeCl_3_·6H_2_O (2 M; Sigma-Aldrich), 90 ml of NaOH (6 M; Sigma-Aldrich), and 10 ml of deionized water were thoroughly mixed in a sealed 250-ml Pyrex bottle. The resulting gel was left undisturbed inside the bottle and aged for 8 days at 100°C. Monodisperse hematite cubes with a size of approximately 800 nm were isolated from the gel by repeated sedimentation and resuspension in deionized water.

Silica-coated cubes were obtained using the Stöber method (48) before the silica coating the hematite cubes was stabilized with a polymer layer of polyvinylpyrrolidone (PVP-10K, Sigma-Aldrich) as described in (49). The PVP-stabilized cubes (6.2 g of a 12 wt % ethanol suspension) were then added to a 2-l round-bottom flask preloaded with 913 ml of ethanol, 66 ml of deionized water, and 10 ml of tetramethylammoniumhydroxide (TMAH 1 wt %, Sigma-Aldrich). The mixture was kept under continuous stirring and sonication at 20°C while 20 ml of a silica precursor solution consisting of tetraethoxyorthosilicate and ethanol (1:1) was added in discrete amounts (2 ml every 20 min). At the end of the additions, the mixture was allowed to react for an additional hour. The resulting silica cubes were washed several times in ethanol via centrifugation.

Monodisperse hexapods were prepared as described in (11). In a typical synthesis, 1 g of polyvinylpyrrolidone (PVP-40K, Sigma-Aldrich) was added to 10 ml of 1-pentanol under constant magnetic stirring at 90°C. When all the PVP was dissolved, 1 ml of ethanol, 200 μl of deionized water, and 200 μl of the hematite stock suspension (6.2 wt % in water) were added to the vial. This mixture was then hand-shaken to homogenize the components before adding 100 μl of aqueous citrate solution (0.18 M). After the addition of citrate, the mixture was placed on a vortex mixer for 1 min. Next, ammonia hydroxide solution (150 μl, 28 wt % in water) was added followed by the addition of 100 μl of tetraethoxyorthosilicate (Sigma-Aldrich) under vortexing. The mixture was then allowed to react undisturbed. After 10 hours, the reaction was quenched by the rapid addition of 30 ml of ethanol/water (1:1) solution. The resulting hexapods were washed several times with ethanol via centrifugation and finally resuspended in deionized water.

### Labeling silica particles

Fifty microliters containing 10 mg of particles were washed with 50% ethanol and then with 95% ethanol. Particles were biotinylated with Silane-PEG-biotin (final concentration 10 mg/ml; PG2-BNSL-3k) in 100 μl of 95% ethanol and incubated on a rotator overnight at 4°C. The particles were then coupled to streptavidin-Alexa Fluor 647 conjugate (Thermo Fisher, S21374) and pHRodo Red avidin (Thermo Fisher, P35362) by first washing three times with distilled water and then adding a mixture of the probes each at 50 μg/ml in 200 μl of PBS. The particles were incubated on a rotator overnight at 4°C. On the next day, the particles were washed three times with distilled water, opsonized with 1:8 anti-streptavidin antibody in 100 μl of PBS, and incubated on a rotator overnight at 4°C. Lastly, the particles were washed three times with PBS, resuspended in 50 μl of PBS and stored at 4°C.

### pH calibration curve

Buffers ranging between pH values of 3.28 and 7.95 (at 37°C) were made from 0.2 M Na_2_HPO_4_ and 0.1 M C₆H₈O₇. ∼3 × 10^5^ stv647-avidin-pHRodo coupled cubes or hexapods in 300 μl of buffer were imaged in an eight-well glass-bottom Ibidi plate (80827).

### Live cell imaging of moDCs

MoDCs were mixed with pre-warmed RPMI-1640 medium without phenol red and incubated for 1 h at 37°C 5% CO_2_. Particles were added in a 1:10 cell/particle ratio for 30, 60, 90, or 120 min and imaged with a Zeiss LSM 800 AiryScan microscope equipped with a 63× 1.4 NA (numerical aperture) oil immersion objective at 37°C.

### Transfection

MoDCs were transfected with the Neon Transfection system (Invitrogen) as previously described (46). Briefly, 10^6^ cells were washed with PBS, resuspended in R buffer (Invitrogen) containing 3 μg of plasmid DNA pEGFP-hGal3 (Addgene #73080; gift from Tamotsu Yoshimori) (23), and electroporated (two pulses, 40 ms, 1000 V). The transfected cells were subsequently plated in an eight-well glass-bottom Ibidi plate (80827) with pre-warmed RPMI-1640 medium without phenol red and incubated for 3 h at 37°C 5% CO_2_. Then 1:10 (cells:particles) particles were added to the wells. Prior to imaging, cells were washed with PBS and fixed with 4% PFA.

### Immunofluorescence

MoDCs were collected from the liquid nitrogen storage, thawed at room temperature, and washed twice with PBS. Then 300,000 cells were plated in an eight-well glass-bottom Ibidi plate (80827) with pre-warmed RPMI-1640 medium without phenol red and incubated for 1 h at 37°C and 5% CO_2_. After incubation, cells were stimulated with either cubes or hexapods in a 1:10 cell:particle ratio for 2 h. Then the cells were fixed with 4% PFA and simultaneously permeabilized and blocked with 0.1% saponin in CLSM buffer for 30–60 min. Thereafter, the cells were treated with a primary antibody in CLSM-Saponin buffer overnight at 4°C followed by secondary antibody staining for 30–60 min at room temperature. The next day, the cells were imaged with a Zeiss LSM 800 microscope equipped with a PlanApochromatic 63× 1.4 NA air immersion objective.

### Electron microscopy and serial sectioning transmission electron microscopy

MoDCs (0.5 × 10^6^) were cultured for 1 H on self-made aluminum (Al) discs for adherence in a 24-well plate cell culture dish in serum-free RPMI supplemented with 2 mM glutamine and 1% antibiotics and antimycotics, followed by hexapod stimulation for 2 h.

Next, the cells were cryo-immobilized by plunging the Al disks rapidly into liquid propane. Al disks with cells were placed on a frozen freeze-substitution medium containing 1% osmium tetroxide, 0.5% uranyl acetate, and 5% water in acetone. The cells were dehydrated and fixed using the rapid freeze-substitution method (50). Samples were embedded in epon and ultrathin 70-nm serial sections were collected on formvar-coated and carbon-evaporated single-hole copper grids. Sections were stained with 2% uranyl acetate and lead citrate solution.

The images were collected with a CM12 (Philips) transmission electron microscope. Using the IMOD software package, and serial electron microscopy images were aligned and rendered in a 3D volume. Using ImageJ, the spacing between the silica surface and the phagocytic membrane for each hexapod was measured at four characteristic geometries (perceived tip, along the rod body, at core-rod bend, and at the core) from which the averaged spacing was estimated.

### Flipper assay

For this, 300,000 moDCs were plated in an eight-well glass-bottom Ibidi plate (80827) with pre-warmed RPMI-1640 medium without phenol red and incubated for 1 h at 37°C 5% CO_2_. Then, Flipper-TR (Cytoskeleton, catalog no. CY-SC020) was added to the wells (1:1000) and incubated overnight at 37°C 5% CO_2_. Afterward, the cells were washed twice with pre-warmed RPMI medium and stimulated with particles in 300 μl of RPMI medium for 1 h. The cells were imaged with a MicroTime 200 microscope (PicoQuant) equipped with an Olympus (100×/1.4 NA) oil immersion objective. Images were acquired using the SymPhoTime 64 software (PicoQuant). Data analysis of the FLIM images was performed using the open-source FLIMfit software (51).

### Statistical analysis

Significance of non-normalized data was evaluated with one-way ANOVA with Holm-Sidak’s post hoc multiple comparisons test or paired *t*-test. Data that did not follow normal distribution were evaluated with paired non-parametric Wilcoxon matched-pairs signed-ranks test. The following p values indicate significance levels: NS, p > 0.05; ∗p ≤ 0.05; ∗∗p ≤ 0.01; ∗∗∗p ≤ 0.001; ∗∗∗∗p ≤ 0.0001.

## Author contributions

Q.Z., T.M.H., and S.S. synthesized the particles. M.N. and T.H. synthesized the iron probe. R.B. performed the electron microscopy. M.V.B., S.B., M.I., and A.B. performed all other experiments. P.G. provided technical support. M.B., S.B., A.B., and G.B. wrote the manuscript with support from M.K., S.S., and S.T. All authors contributed to the article and approved the submitted version.

## Primary data

https://doi.org/10.5281/zenodo.5654491.

## Supporting material

Document S1. Figures S1–S5

Document S2. Article plus supporting material
