# Supplementary material for: Irregular particle morphology and membrane rupture facilitate ion gradients in the lumen of phagosomes
Source: Biophys Rep (N Y). 2022 Aug 11;2(3):100069. doi: 10.1016/j.bpr.2022.100069 (PMC9680789; doi:10.1016/j.bpr.2022.100069)
Supplement: Document S1. Figures S1–S5 [file mmc1.pdf]

Biophysical Reports, Volume *2*

Supplemental information

Irregular particle morphology and membrane rupture facilitate ion gradients in the lumen of phagosomes

Maksim V. Baranov, Melina Ioannidis, Sami Balahsioui, Auke Boersma, Rinse de Boer, Manoj Kumar, Masato Niwa, Tasuku Hirayama, Qintian Zhou, Terrence M. Hopkins, Pieter Grijpstra, Shashi Thutupalli, Stefano Sacanna, and Geert van den Bogaart

SUPPLEMENTARY INFORMATION

Irregular particle morphology and membrane rupture facilitate ion gradients in the lumen of phagosomes

Baranov M.V.[1], Ioannidis, M.[1], Balahsioui S.[1], Boersma A.[1], de Boer R.[1], Kumar M.[2], Niwa M.[3], Hirayama T.[3], Zhou Q.[5], Hopkins T.M.[5], Grijpstra, P.[1], Thutupalli S.[2,4], Sacanna S.[5], van den Bogaart G.[1,6]

1. Department of Molecular Immunology, Groningen Biomolecular Sciences and Biotechnology Institute, University of Groningen, Groningen, Netherlands
2. Simons Centre for the Study of Living Machines, National Centre for Biological Sciences, Tata Institute of Fundamental Research, Bangalore, India
3. Laboratory of Pharmaceutical and Medicinal Chemistry, Gifu Pharmaceutical University, 1–25–4, Daigaku-nishi, Gifu 201–1196, Japan
4. International Centre for Theoretical Sciences, Tata Institute of Fundamental Research, Bangalore, India
5. Molecular Design Institute, Department of Chemistry, New York University, New York, NY, United States
6. Department of Medical Biology and Pathology, University Medical Center Groningen, Groningen, Netherlands

Contents:

-5 supplementary figures

-4 supplementary movie legends

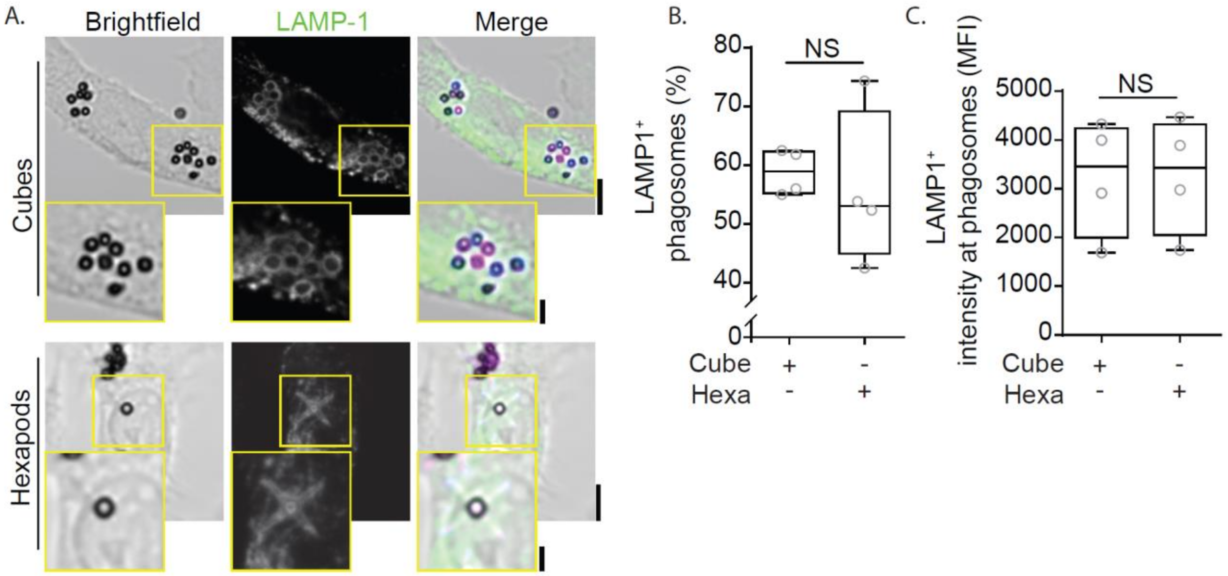

Supplementary figure 1. (A) Representative confocal micrograph showing immunolabeled LAMP-1 (green in merge) recruitment to phagosomes containing cubes or hexapods internalized by monocyte-derived DCs (moDCs). Scale bars: main images, 5 µm, insets, 2 µm. **(B)** Quantification of panel A. (mean per donor$\pm$SEM; ~156 cubes/donor and ~97 hexapods/per donor; 4 donors). **(C)** Quantification of LAMP1 intensity at phagosomes from panel A. (mean per donor$\pm$SEM; ~86 cubes/donor and ~ 64 hexapods/per donor; 4 donors). Statistics: paired t-test. NS: not significant.

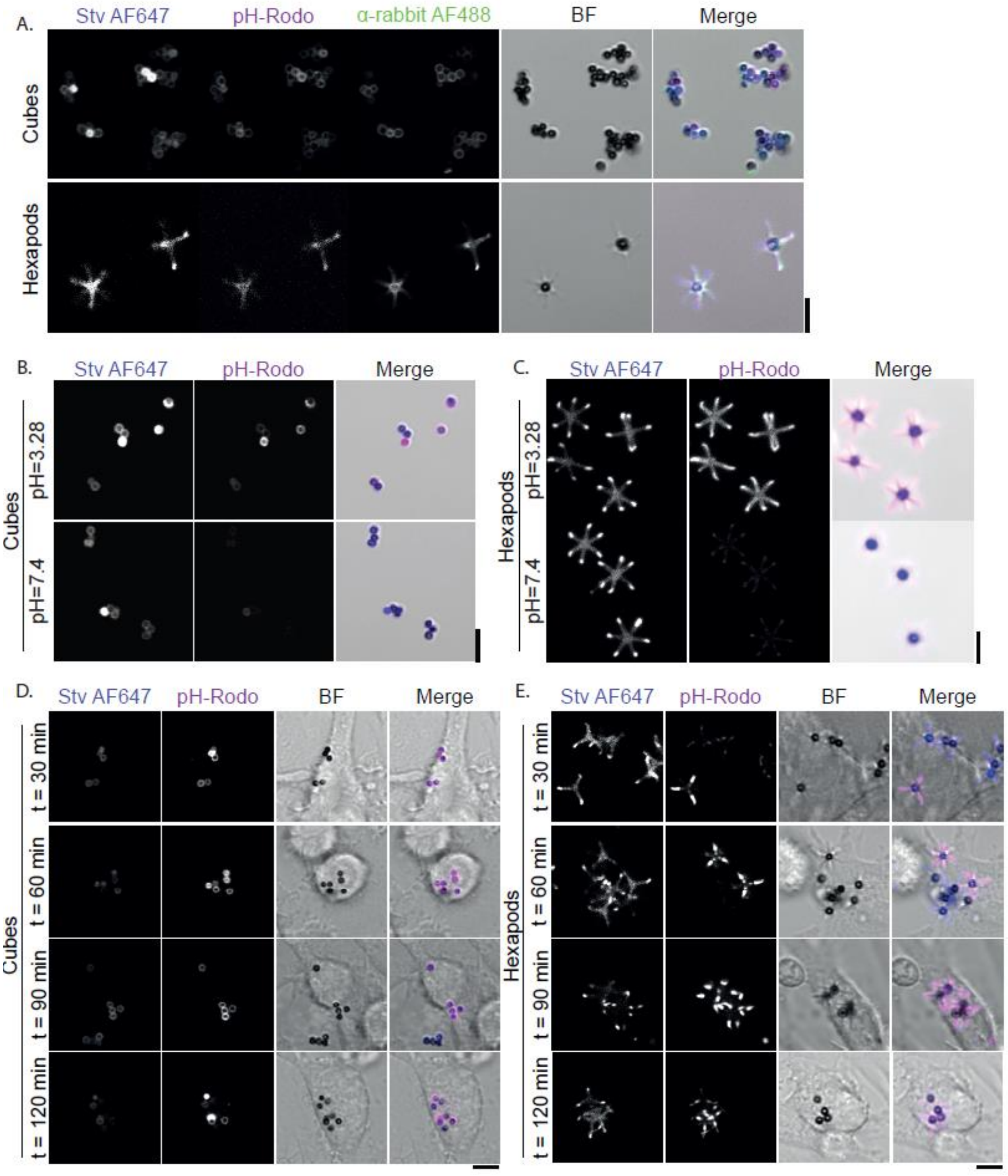

Supplementary figure 2. (A) Representative confocal micrograph of particles imaged in PBS, showing the efficiency of silica surface functionalization with biotin-silane and subsequent coating with Streptavidin-Alexa Fluor 647 (Stv AF 647, blue in merge) and Avidin-phRodo-Red (pH-Rodo, magenta), followed by opsonization with Anti-Streptavidin-rabbit-antibody to facilitate the Fc-mediated uptake, detected with α-rabbit secondary antibody (α-rabbit AF488, green). BF: bright-field. **(B)** Representative confocal micrograph of cubes at pH~3.28 and pH~7.4 (the buffers were also used in Fig. 2C). **(C)** Confocal

micrograph of hexapod fluorescence in buffers with two marginal pH~3.28 or pH~7.4 (the buffers were also used in Fig. 2D). **(D)** Representative confocal micrographs of monocyte-derived DCs (moDCs) exposed to cubes for the indicated time points (quantified in Fig. 2E). **(E)** same as panel D but for hexapods (quantified in Fig. 2E). Scale bars: 5 µm.

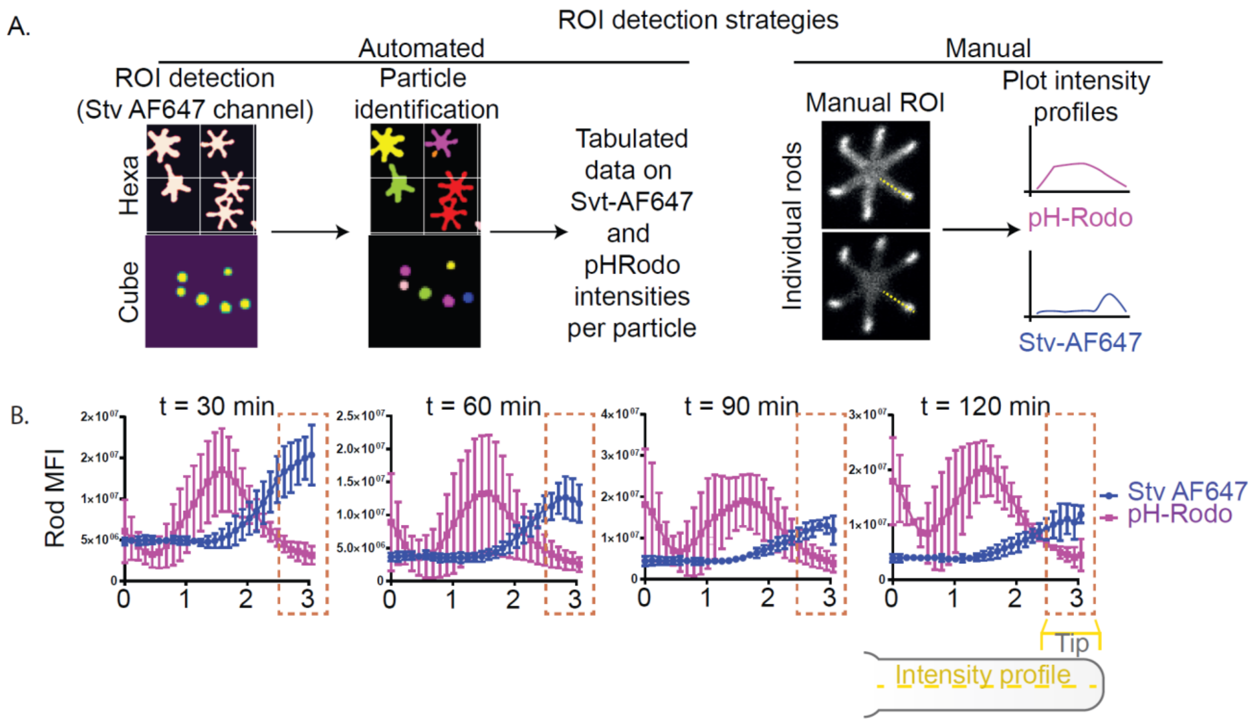

Supplementary figure 3. (A) Region of interest (ROI) detection for particles. Left-hand panel: automated analysis with a Python script. Right-hand: manually plotted fluorescence intensity profile over the length of each rod from the hexapod core to the tip (yellow line). **(B)** Averaged plot profiles per rod from hexapods in cells from Suppl. Fig.2E (mean±SD; ~48 rods/per donor/time point; 4 donors). Red dashed-boxes show the 0.5 µm hexapod tip analyzed in Fig. 2F.

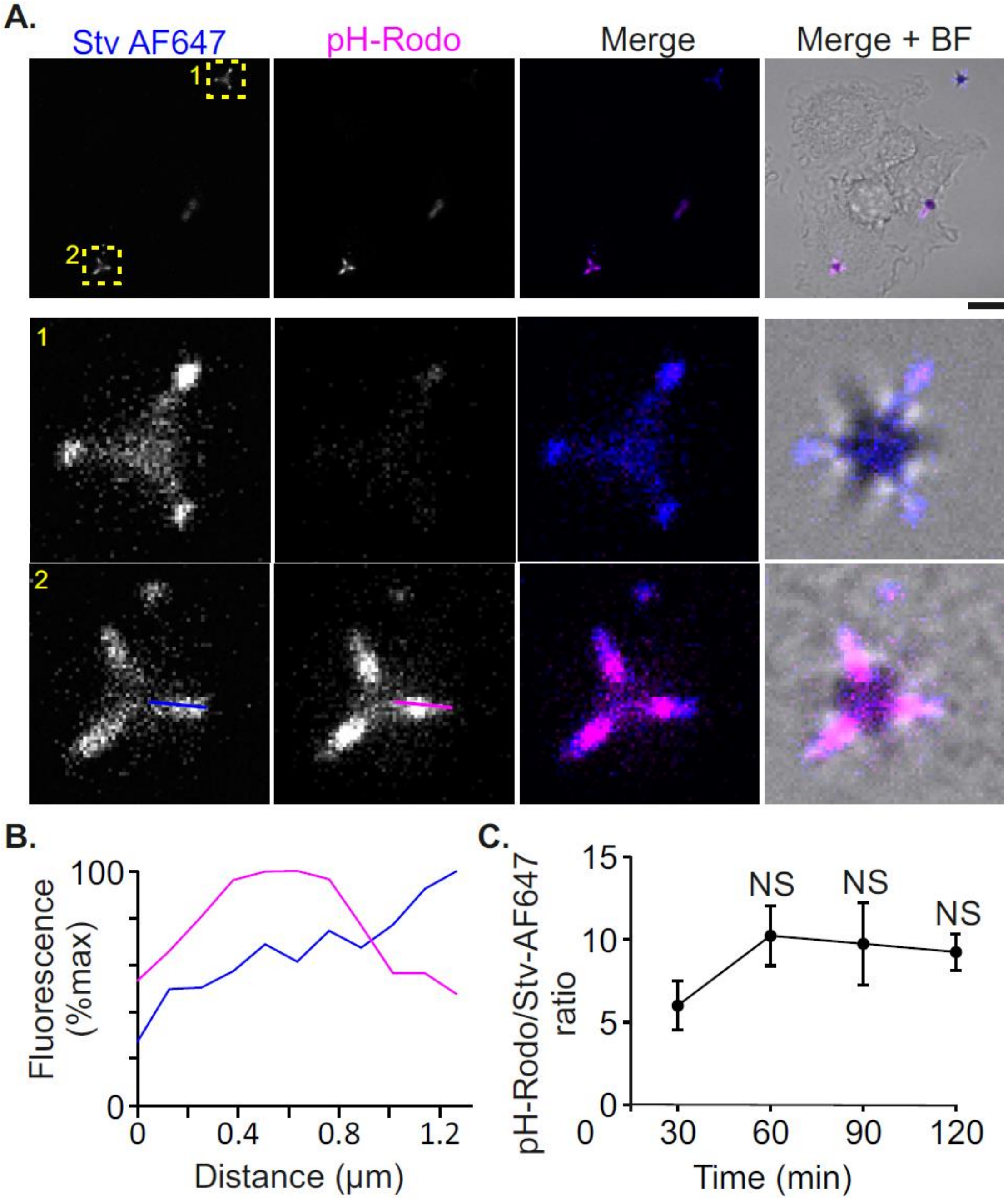

Supplementary figure 4. Proton gradient in monocyte-derived macrophages. Representative confocal micrograph of monocyte-derived macrophages pulsed with hexapods labeled with Streptavidin-Alexa Fluor 647 (Stv AF647, blue in merge) and avidin-pHRodo (magenta). Region of interest #1: Non-ingested hexapod (note the low pHRodo signal). Region of interest #2: Ingested hexapod (note the elevated pHRodo signal). BF: bright-field. Scale bar: 10 µm. **(B)** Intensity plot profiles of Stv AF647 (blue) and avidin-pHRodo (magenta) as indicated in panel A. **(C)** Ratio of pH-Rodo over Stv AF647 signals for phagosomes carrying hexapods in macrophages from 3 donors pulsed for the indicated times (mean±SEM based on >15 particles/per donor/time point). Statistics: 1-way ANOVA with Bonferroni's multiple comparison test compared with time = 30 min. Note that signals differ because the particles used for this experiment were from a different badge than those used for the other experiments.

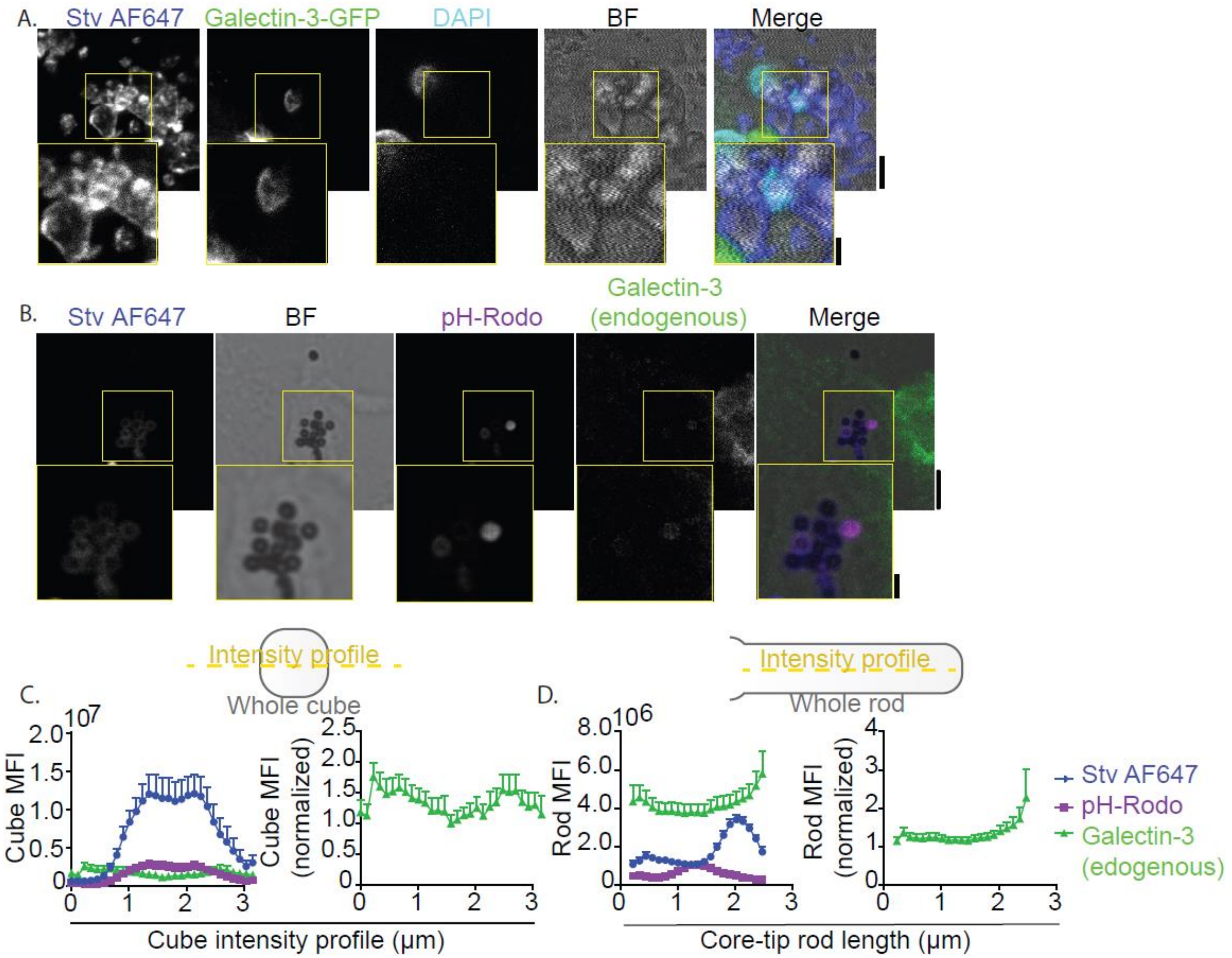

Supplementary figure 5. (A) Representative confocal micrograph showing overexpression of galectin-3-GFP (green) in monocyte-derived DCs (moDCs) followed by stimulation with silica chips for 60 min. BF: bright-field. Blue: DAPI. **(B)** Representative confocal micrograph showing immunolabelling of endogenous galectin-3 (green in merge) in monocyte-derived DCs (moDCs) stimulated with cubes for 120 min. Scale bars: main images 5 µm, insets 2 µm. **(C)** Averaged intensity cross-section per cube (left-hand) and normalization to cytosolic background (right hand). (mean$\pm$SEM; 28 cubes). **(D)** Same as panel C but intensity plot profiles over the length of the rods of the hexapods (mean$\pm$SEM; 42 hexapods).

Supplementary Movie 1. Live imaging of hexapod uptake by moDCs over 5 hr. Cyan: DAPI. Green: hexapod-labeled stv-647.

Supplementary Movie 2. Three-dimensional reconstruction of confocal imaging of a hexapod (stv-647, green) internalized by a monocyte-derived dendritic cell. Red: F-actin. Gray: DAPI.

Supplementary Movie 3. Three-dimensional reconstruction of an electron microscopy serial section images of a hexapod (red) internalized by a monocyte-derived dendritic cell. The phagosome is surrounded by intracellular organelles: mitochondria (green), endo/lysosomes (yellow), and nucleus (blue).

Supplementary Movie 4. Three-dimensional reconstruction of an electron microscopy imaging of a hexapod (red) internalized by a monocyte-derived dendritic cell. The phagosome is surrounded by intracellular organelles: mitochondria (green), endo/lysosomes (yellow), and plasma membrane (purple). Note the incomplete internalization of the particle.
